# Epidemiological and Histological Characteristics of Differentiated Thyroid Carcinoma: A Case of the Department of Nuclear Medicine at Ibn Rochd Hospital, Casablanca, Morocco

**DOI:** 10.7759/cureus.52033

**Published:** 2024-01-10

**Authors:** Hajar Tabiti, Amal Guensi, Karima Bendahhou

**Affiliations:** 1 Laboratory of Cellular and Molecular Pathophysiology of Inflammation, Degeneration, and Oncology, Hassan II University of Casablanca, Casablanca, MAR; 2 Department of Nuclear Medicine, Ibn Rochd Hospital, Casablanca, MAR; 3 Department of Epidemiology and Public Health, Ibn Rochd Hospital Casablanca Cancer Registry, Casablanca, MAR

**Keywords:** differentiated thyroid cancer, epidemiological profile of dtc, histological profile of dtc, variants of papillary thyroid carcinoma, observational cross-sectional study

## Abstract

Background: Thyroid carcinoma (TC) represents the most frequent type of endocrine cancers, with its incidence steadily increasing worldwide. Our study aimed to describe the epidemiological and histological characteristics of differentiated thyroid carcinoma (DTC) at the Department of Nuclear Medicine in Ibn Rochd University Hospital, Casablanca, Morocco.

Methods: This was a cross-sectional study of DTC cases treated between 2004 and 2012 in the Department of Nuclear Medicine at Ibn Rochd University Hospital. We retrospectively reviewed medical records at this department, focusing on sociodemographic characteristics, such as age, gender, geographic origin, family history of cancer, and clinical information related to tumor features, including histological type, tumor size, and multifocality. The data were statistically analyzed using the jamovi 2.3.17 software (released September 2022, the jamovi project, retrieved from https://www.jamovi.org), considering the characteristics of the variables.

Results: The findings revealed that 89% of the patients were females, and 78.7% were under the age of 55, ranging from 14 to 85 years. Married status represented 75.25% of our cases. Personal history of cancer and a family history of thyroid carcinoma were present in 0.9% and 1.17%, respectively. Concerning histological characteristics, the main histological type was papillary thyroid carcinoma (PTC) at 93%. Within these 1,283 cases, the follicular variant was the most frequent (42.89%). In addition, the tumor size was less than 2 cm in 43.80%, and it was encapsulated in 21.60%. Moreover, we staged our data according to the 8th edition of the American Joint Committee on Cancer staging system, revealing that at the time of diagnosis, 94.13% were in stage I.

Conclusion: This study provides an overview of the epidemiological and histological characteristics of DTC in Morocco. The findings highlight the diversity and differences between clinical presentation and epidemiological profile in Moroccan patients, contributing to a better understanding of the disease and facilitating adapted management.

## Introduction

Thyroid carcinomas (TCs) are the most common form of endocrine cancer, and they have the potential to develop at any age. Their occurrence has consistently risen globally over the past three decades [1]. In the 2020 GLOBOCAN report, the age-standardized incidence rate of TC was 10.1 per 100,000 women and 3.1 per 100,000 men. Incidence rates show that women are being more frequently affected than men across worldwide. However, the highest incidence had been recorded in the Federated States of Micronesia and French Polynesia (18.5 per 100,000 women), North America (18.4 per 100,000), and East Asia (17.8 per 100,000, with South Korea reaching 45 per 100,000) [2]. In Morocco, 1,924 cases of TC were recorded between 2013 and 2017 in the Greater Casablanca Region. Approximately, this represents 8% of all cancers in both sexes and 11.3% of cancers in women. The crude incidence rate was 15.2 in women versus 2.6 per 100,000 in men. This number places the TC in the second most common cancer in women after breast cancer [3].

The growing incidence of TC is almost entirely due to the differentiated thyroid carcinoma (DTC), most common thyroid malignancy, especially papillary thyroid carcinoma (PTC), which occurs more than 70% of thyroid malignancies. The classification of the disease is important because it determines the therapeutic approach. The most commonly utilized classification for TCs is the one recommended by the World Health Organization (WHO). DTC is divided into three major types: PTC, follicular TCs (FTCs), and Hurthle cell carcinoma (HCC). DTC’s prognosis depends on the histological type, size, and stage, but in general, it is good (over 90%) [4-6]. In fact, the epidemiological studies of cancer in general and in TC contribute to better management of patients. Therefore, the purpose of this study is to describe the epidemiological and histopathological aspects of DTC in the Department of Nuclear Medicine at Ibn Rochd Hospital, Casablanca, Morocco.

## Materials and methods

Patients and methods

Study Design

This is a cross-sectional study. We investigated retrospective cases of DTC of follicular origin managed within the Department of Nuclear Medicine in Ibn Rochd University Hospital over a comprehensive eight-year period from 2004 to 2012. This research was ethically conducted with the approval of the Ethics Committee for Biomedical Research of Casablanca, under Order No. 02/2022. Furthermore, the study received explicit approval from the Ethics Committee of Ibn Rochd University Hospital, indicated by Order no. 14/22.

Study Population

All patients were Moroccans, and for each case, data regarding age, gender, circumstance of discovering, tumor size at diagnosis, and histology were collected. The inclusion criteria encompassed cases of DTC that underwent treatment between 2004 and 2012. Notably, patients with the medullary thyroid carcinoma and those with primary tumors metastasizing to the thyroid were excluded from the study.

Data Collection and Statistical Analysis

Data were meticulously extracted from medical records. Our statistical analysis utilized the jamovi 2.3.17 software (released September 2022, the jamovi project, retrieved from https://www.jamovi.org), involving the calculation of percentages for qualitative variables along with their confidence intervals. In addition, means for quantitative variables were determined, accompanied by standard deviation measurements.

## Results

A total of 1,366 of cases of DTC was recorded during the period between 2004 and 2012. Most of the cases were female (1,223 (89.6%)) with a female-to-male ratio of 8.5. The average age was 44 years, with extremes ranging from 14 to 85 years and a standard deviation of 12.8. Moreover, 1,079 patients (79%) are under the age of 55 years (confidence interval (CI) 5%: 67.1-80.5). In terms of marital status, 917 individuals were married (75%), while 214 patients were single (17.33%). Regarding geographic origin, 828 patients hailed from coastal areas (51.60%) (Table 1).

**Table 1:** Summary of the epidemiological features of our patients (n = 1,366)

Variable	Frequency	Percentage %
Gender		
Female	1,223	89.6
Male	143	10.4
Age (n = 1,360)		
<55 years	1,079	78.70
≥55 years	289	21.20
Marital status (n = 1235)		
Single	214	17.33
Married	917	75.25
Divorced	35	2.83
Widowed	69	5.59
Geographical origin (n = 1,344)		
Coastal areas	828	61.60
Noncoastal areas	516	38.39
History of thyroid diseases		
Yes	5	0.36
Non	1,361	99.63
History of cancers		
Yes	9	0.90
No	1,357	99.34
Family history of thyroid diseases		
Yes	232	17.00
No	1,134	83.00
Family history of TC		
Yes	16	1.17
No	1,350	98.83

Five patients had thyroid diseases before having TC. In addition, a history of cancer was present in nine cases with breast cancer and cervical cancer as most frequent locations. A family history of TC was found in 16 cases (1.17%) and of thyroid disease in 125 cases with the highest frequency of goiter (17%). None of our cases had antecedents of cervical radiation (Table 1).

The circumstance of discovery was marked by nodules in 1,049 patients (86.10%), and it was associated to pressure signs in 12% of cases and with the signs of dysthyroidism in 10.4%. In addition, at the time of diagnosis, 30 patients exhibited cervical lymph nodes, while eight patients exclusively showed distant metastasis (in the brain, lungs, and bones) (Table 2).

**Table 2:** Distribution of cases based on the circumstances of discovering differentiated thyroid carcinoma

Variable	Frequency	Percentage %
Circumstance of discovery (n = 1,219)		
Thyroid nodule	1,049	86.10
Accidental discovery	170	13.90
Pressure sings (n = 1,163)		
Yes	140	12.00
No	1,023	88.00
Dysthyroidism (n = 1,168)		
Yes	122	10.4
No	1,046	89.60
Cervical lymph node (n = 1,163)		
Yes	30	2.58
No	113	97.42
Distant metastasis at diagnosis		
Yes	8	0.58
No	1,358	99.41
Total: 1,366		

Clinical and histological characteristics

All our patients (1,366 in total) underwent a total thyroidectomy, with 73% of cases undergoing the procedure immediately. PTC emerged as the predominant histological type (n = 1,273, 93%) (CI 5%: 91.73-94.41), followed by FTC in only 88 patients. Among the 1,273 cases of PTC, 241 (19%) were classified as papillary microcarcinoma. The follicular variant constituted 42.89% of PTC variations (CI: 40.19-45.62). Furthermore, an undifferentiated component was identified in only 14 patients (Table 3).

**Table 3:** Distribution of DTC cases based on clinical and histological characteristics DTC: differentiated thyroid carcinoma, PTC: papillary thyroid carcinoma, FTC: follicular thyroid carcinoma, HCC: Hürthle cell carcinoma

Variable	Frequency	Percentage %	CI
Thyroidectomy			
Tow time	362	26.5	24.20-28.9
One time	1,004	73.5	71.10-75.80
Histological type			
PTC	1,273	93.19	91.73-94.41
FTC	88	6.44	5.25-7.87
HCC	5	0.36	0.15-0.85
PTC variants			
Follicular	546	42.89	40.19-45.62
Conventional	450	35.35	32.77-38.01
Papillary microcarcinoma	241	18.93	16.87-21.17
Trabecular	18	1.31	0.83-2.07
Oncocytic	15	1.17	0.71-1.93
Insular	2	0.11	
Tall cell	1		
Undifferentiated component (n = 1,364)			
Yes	14	1.03	0.61-1.72
No	1,350	98.97	98.28-99.39
Tumor size (n = 1,070)			
<2 cm	469	43.80	40.90-46.80
2 to 4 cm	456	42.60	39.7-45.60
>4 cm	145	13.60	11.6-15.70
Encapsulated (n = 1,353)			
Yes	292	21.60	19.50-23.90
No	1,061	78.40	76.10-80.50
Unilateral (n = 1,354)			
Yes	1,107	81.8	79.60-83.70
No	247	18.2	16.30-20.40
Focality (n = 1,356)			
Unifocal	967	71.3	68.80-73.70
Bifocal	206	15.2	13.40-17.20
Multifocal	183	13.5	11.80-15.40
Nodular capsule invasion (n = 1,357)			
Yes	193	14.2	12.50-16.20
No	1,164	85.8	83.80-87.50
Vascular embolus (n = 1,358)			
Yes	84	6.19	5.02-7.59
No	1,274	93.81	92.41-94.98
Extrathyroidal fraction (n = 1,357)			
Yes	73	5.38	4.30-6.71
No	1,284	94.62	93.29-95.70
Lymph node dissection (n = 1,357)			
Yes	126	9.29	7.85-10.90
No	1,231	90.71	89.05-92.10
Result of Lymph node dissection (n = 115)			
Positive	77	67.00	57.90-74.90
Negative	38	33.00	25.10-42.10
Stage (n = 1,176)			
I	1,107	94.13	92.64-95.33
II	54	4.59	3.53-5.94
III	8	0.68	0.34-1.33
IV	7	0.51	0.21-1.05
Total: 1,366			

The tumor size was less than 2 cm in 469 patients (43.80%, CI 5%: 40.9-46.8). Encapsulated tumors were observed in 292 patients (21.60%). The disease manifested unilaterally in 1,107 cases (82%, CI 5%: 79.6-83.7) and multifocally in 183 cases. Regarding local invasion, nodular capsule invasion and extrathyroidal extension were present in 14% and 5.38% of cases, respectively. Vascular embolus accounted for 84 cases (6.19%) (Table 3).

Lymph node dissection was performed in 126 cases (9.29%), with positive findings in 77 cases. Patients were categorized according to the 8th edition of the American Joint Committee on Cancer staging system for TC (AJCC-8) at the time of diagnosis, facilitating statistical analysis. The results indicated that 94.13% were in stage I (CI 5%: 92.64-95.33) (Table 3).

## Discussion

Our study focused on the epidemiological and histopathological characteristics of DTC over an eight-year period (from 2004 to 2012) in the Department of Nuclear Medicine at Ibn Rochd Hospital. A total of 1,366 cases were recorded, averaging 170 cases per year. The majority of our cases were female (89.6%), with a female-to-male ratio of 8.55. This prevalence of females aligns with observations in the literature, both nationally and internationally. A study conducted at Ibn Sina Hospital in Rabat by Ben Raïs Aouad et al. showed a high proportion of TC in women with a sex ratio of 3.5 [7]. Internationally, a study in China in 2017 found that 76.9% of DTC cases were female, confirming the female trend in thyroid disease [8].

Thyroid body tumors affect all age groups, with a frequency that increases with age, as described in the literature [9]. The mean age at diagnosis in our survey was 44 years, ranging from 14 to 85 years. These results are similar to those found in the literature. For example, a study in the United States on tumor characteristics at the time of diagnosis reported that 40% of cases were diagnosed between the ages of 40 and 59 years [10]. In our survey, marital status was characterized by 75.25% of cases being married and 17.33% being single. In addition, 61% of our patients came from coastal areas with a known high iodine intake due to the availability of seafood. Several epidemiological studies suggest that iodine intake could be related to the incidence of TC. High iodine intake was identified as a significant risk factor for the occurrence of BRAF mutation in the thyroid gland, which is involved in PTC [11].

In our study series, the thyroid nodule was the most frequent clinical presentation, accounting for 86.10% of cases. It was associated with pressure signs in 12% and dysthyroidism in 10.4%. Tis are recognized by the presence of a cervical mass, either as a nodule or as part of a multinodular goiter. This nodule may be palpable on clinical examination or visible on cervical ultrasound. Several studies have demonstrated that the thyroid nodule is a common presentation for TC. For instance, Zuberi et al. in a study in Pakistan found that 59% of patients with TC presented with a "thyroid mass," and Touati et al. found that 76% of cases leading to the diagnosis of TC were associated with a cervical mass [1,12].

Surgery is the mainstay of treatment for TC, and the extent of the surgery can be categorized as total thyroidectomy (TT) or lobectomy (LT). In our survey, all patients underwent TT, either in a single time (73.5%) or as a LT extended to TT. Lymph node dissection was performed in 9.29% of cases and was positive in 67%. Regarding tumor size, 43.80% of the study population had tumors smaller than 2 cm, consistent with the results of Lim et al. and Boukheris et al., who found that 54% and 48% of their study populations had tumors <2 cm, respectively [13,14]. However, these results disagree with those of Ben Raïs Aouad et al., who found that 70% of cases in their study had tumors >2 cm. This contradiction can be explained by the increase in the incidence of small tumors, as described in the literature, due to the development of diagnostic methods [7].

The main histological type of DTC in our study was PTC, accounting for 93%, followed by FTC with 6.44%. This result confirms that PTC is the most frequent thyroid tumor compared to other histological types. Trimboli et al. demonstrated that the rate of PTC reaches 87.8% in 500 cases of TC [14]. Furthermore, follicular variants accounted for 42.89% of PTC, followed by the conventional type with 44%. These differences in the percentage of conventional and follicular variants of PTC have also been described by Sassolas et al., with 28% and 30%, respectively [15].

According to the results of the anatomopathological study, the disease in our series was unilateral in 81.8%, unifocal in 71.3%, and non-encapsulated in 78.4%, consistent with the literature. In 2021, Huang et al. found in a series of 484 cases that unilateral tumors were observed in 82%, and they were unifocal in 88%. In addition, the study by Sciuto et al. revealed that multifocality was present in only 22.8%, compared to 77% unifocal tumors [16,17]. For local invasion, nodular capsule invasion was present in 14%, thyroid capsule invasion in 5.38%, and vascular embolus accounted for 6.19%. These pathological features align with other studies. M’hamed et al. reported that vascular invasion, nodular capsular invasion, and extra-thyroidal fraction were detected in 7%, 35.2%, and 16.9%, respectively. However, the study by Prak et al. showed a higher percentage of extra-thyroidal extension and vascular invasion, reaching 62.2% and 68.8%, respectively [18,19].

The classification of the disease is important as it determines the therapeutic approach. The most commonly used classification for staging TCs is the 8th edition of the AJCC staging system for TC (AJCC-8). In our patient population, 94.13% were in stage I at diagnosis, consistent with the results of Zhu et al., who found 78% of patients at stage I in 1,970 cases [8].

There are some limitations to this work. First, it is a retrospective analysis from a single department in a public hospital, catering to patients with low incomes. Second, we lack information about the diagnostic process before surgery, which could be useful to better interpret the results. However, this study is one of the few studies based on a large enough sample, and even though it is from a single department, it covers a significant portion of the south of Morocco, which includes a substantial part of the Moroccan population.

## Conclusions

Our study provides insights into the landscape of DTC in Morocco, revealing a notable increase in the frequency of PTC as the primary histological type. Consistent with the existing literature, our findings highlight that females and individuals under 55 years old are the most affected categories by the disease. Notably, a significant proportion of our cases (44%) involve small tumor sizes.

This observation contributes to our understanding of the continuous increase in TC incidence in our country. We attribute this rise, in line with numerous other studies, to overdiagnosis resulting from the heightened use of advanced imaging modalities. Consequently, there is a pressing need for research on patient survival that can help predict which cancers are likely to pose significant issues, ultimately aiding in avoiding unnecessary interventions.
